# First species record of *Strigea falconis* Szidat, 1928 (Trematoda, Strigeidae) from gyrfalcon *Falco rusticolus* in Iceland—pros and cons of a complex life cycle

**DOI:** 10.1007/s00436-024-08161-w

**Published:** 2024-03-04

**Authors:** Anna Faltýnková, Damien Jouet, Ólafur Karl Nielsen, Karl Skírnisson

**Affiliations:** 1 Department of Forest Ecology, Faculty of Forestry and Wood Technology, Mendel University in Brno, Zemědělská 3, Brno, 613 00 Czech Republic; 2 ESCAPE UR7510, USC ANSES PETARD, Faculty of Pharmacy, University of Reims Champagne-Ardenne, 51 Rue Cognacq-Jay, 51096 Reims Cedex, France; 3 Icelandic Institute of Natural History, Garðabær, Iceland; 4 Laboratory of Parasitology, Institute for Experimental Pathology, Keldur, University of Iceland, IS-112 Reykjavík, Iceland

**Keywords:** Digenea, Trematoda, Raptors, Gyrfalcon, Life cycle, Nuclear and mitochondrial DNA

## Abstract

*Strigea falconis* is a common parasite of birds of prey and owls widely distributed in the Holarctic. We aimed to characterise *S. falconis* from Iceland via integrative taxonomic approach and to contribute to the understanding of its circulation in the Holarctic. We recovered adult *S. falconis* from two gyrfalcons (*Falco rusticolus*) collected in 2011 and 2012 in Iceland (Reykjanes Peninsula, Westfjords) and characterised them by morphological and molecular genetic (D2 of rDNA, *cox*1, ND1 of the mDNA) methods. We provide the first species record of *S. falconis* in Iceland which to the best of our knowledge is its northernmost distributional range. The presence of *S. falconis* in Iceland is surprising, as there are no suitable intermediate hosts allowing completion of its life cycle. Gyrfalcons are fully sedentary in Iceland; thus, the only plausible explanation is that they acquired their infection by preying upon migratory birds arriving from Europe. Our data indicate that the most likely candidates are Anseriformes and Charadriiformes. Also, we corroborate the wide geographical distribution of *S. falconis*, as we found a high degree of similarity between our haplotypes and sequences of mesocercariae from frogs in France and of a metacercaria from *Turdus naumanni* in Japan, and adults from *Buteo buteo* and *Circus aeruginosus* from the Czech Republic. The case of *Strigea falconis* shows the advantages of a complex life cycle and also depicts its pitfalls when a parasite is introduced to a new area with no suitable intermediate hosts. In Iceland, gyrfalcons are apparently dead-end hosts for *S. falconis*.

**Supplementary Information:**

The online version contains supplementary material available at 10.1007/s00436-024-08161-w.

## Introduction

*Strigea falconis* Szidat, 1928 of the family Strigeidae Railliet, 1919 is a trematode parasite widely distributed in the Holarctic region, reported from a wide range of birds of prey and owls (Bykhovskaya-Pavlovskaya 1962; Dubois 1968; Sudarikov 1959, 1984; Krone and Streich 2000; Heneberg et al. 2018). The systematics of the family Strigeidae is still quite unsettled, and DNA sequences of *S. falconis* and other members of the family have been scarce. So far, Heneberg et al. (2018) provided the most comprehensive analysis of European strigeids applying the approach of integrative taxonomy. Most recently, there have been attempts to tackle the phylogenetic relationships of the species of the genus *Strigea* Abildgaard, 1790 and related genera (López-Jiménez et al. 2023), which together with Heneberg et al. (2018) confirmed the status of *S. falconis* as a well-defined species.

*Strigea falconis* has a complex, four-host life cycle involving several intermediate and paratenic hosts (Bykhovskaya-Pavlovskaya 1962; Dubois 1968; Sudarikov 1959, 1984). The pulmonate snail *Planorbis planorbis* (L.) (Gastropoda, Planorbidae) is used as the first intermediate host (Odening 1964); the second intermediate hosts are tadpoles and frogs harbouring the unencysted stage of mesocercaria; as the third intermediate hosts, amphibians, snakes, birds and mammals are used which harbour the encysted stage of metacercariae, and some of these hosts are considered acting as paratenic hosts (Sudarikov 1959, 1984; Odening 1967; Dubois 1968, Niewiadomska (2002); Sitko et al. 2006). Concerning definitive hosts, Heneberg et al. (2018) confirmed by DNA sequence analysis, that *S. falconis* is using primarily birds of prey, particularly *Buteo buteo* (L.) and *Circus aeruginosus* (L.) (the type-host), less frequently species of *Falco*, while owls are considered as rare definitive hosts. Krone and Streich (2000) noted that *S. falconis* is the most often recorded trematode in birds of prey in Europe, and indeed, there are numerous reports regarding it as a common, generalist parasite of birds of prey (see Okulewicz et al. 1993; Sitko 1998; Sanmartín et al. 2004; Sitko et al. 2006; Santoro et al. 2010; Jantošková and Halán 2014; Komorová et al. 2016, 2017; Zafra et al. 2022). Dubois (1968) in his compilation provided a long list of definitive hosts (Accipitriformes, Falconiformes and Strigiformes) from the Holarctic, including Great Britain, where *S. falconis* was recorded from *Falco rusticolus islandus* Brünnich, which is to the best of our knowledge the only record of *S. falconis* from a gyrfalcon (see Baylis 1939).

The gyrfalcon, *Falco rusticolus* L., has a circumpolar distribution, and its populations are found breeding in the high and the sub-Arctic (Cramp and Simmons 1980; Koskimies and Sulkava 2011; Nielsen 2011). In Iceland, the estimated size of the gyrfalcon population is 3–400 breeding pairs (Icelandic Institute of Natural History (IINH), 2000), and the birds are fully sedentary (Nielsen and Cade 1990a). The gyrfalcons are prey-specialists relying on willow and rock ptarmigan (*Lagopus lagopus* (L.), *L. muta* (Montin)); however, during summer, ptarmigan is supplemented by alternative prey, mainly waterfowl (Anseriformes), waders (Charadriiformes), auks (Alcidae) and mammals (hares, microtine rodents) (Bengtson 1971; Nielsen 2003, 2011; Nielsen and Cade 1990b). Given the range of prey, gyrfalcons might be potential hosts for several trematodes; however, the records have so far been quite sporadic (Clausen and Gudmundsson 1981; Christensen et al. 2015). As mentioned above, Baylis (1939) recorded *S. falconis* from a gyrfalcon in Great Britain (Scotland), and Dubois and Rausch (1965) reported another strigeid, *Strigea macropharynx* Dubois & Rausch, 1965, and a diplostomid, *Neodiplostomum spathula banghami* Penrod, 1947, from a gyrfalcon in North America, Alaska. In Iceland, Clausen and Gudmundsson (1981) recorded *Plagiorchis elegans* (Rudolphi, 1802) from gyrfalcons; and Christensen (2013) and Christensen et al. (2015) found few trematode species in a large-scale sample of gyrfalcons: *Cryptocotyle concava* (Creplin, 1825), *C. lingua* (Creplin, 1825), *Cryptocotyle* sp., *Levinseniella propinqua* Jägerskiöld, 1907, *Microphallus pygmaeus* (Levinsen, 1881), *P. elegans* and *Strigea* sp., the latter not being further identified. Except for *Strigea* sp., the authors assumed that the trematodes might have been accidentally ingested with prey and left the question open if gyrfalcons are used as proper definitive hosts by most of these species.

In this study, our aim was to identify via an integrative taxonomic approach these *Strigea* spp. isolated from gyrfalcons in Iceland and to contribute to the understanding of the reason for their presence in this host at these latitudes and their circulation within the Holarctic zone. This new species record for Iceland is the more surprising, as the intermediate hosts (*P. planorbis*, amphibians, reptiles) necessary for completion of the four-host life cycle of *S. falconis* are absent in Iceland, while gyrfalcons are sedentary (Nielsen and Cade 1990a). Below, we provide data on morphology combined with genetic data and information on the geographical distribution and the life cycle of *S. falconis* including a possible explanation for the record from Iceland.

## Material and methods

### Sampling and processing of bird hosts and parasite material

This study, focused on digenetic trematodes of *Strigea* spp., benefited from a collection of carcasses of gyrfalcons, *Falco rusticolus*, provided by the Icelandic Institute of Natural History in Garðabær, and which was the basis for a thesis examining their endo- and ectoparasites (Christensen 2013; and Christensen et al. 2015). In total, 25 gyrfalcon carcasses from Iceland were examined for helminth endoparasites (for details see Christensen 2013), and those specimens preliminarily identified as *Strigea* spp. were set aside for the present study.

The two gyrfalcons infected with *Strigea* spp. were collected in the western part of Iceland: (i) a 2nd cal. year male (id. N. Fr-12–39) was found dead on 31 May 2011 at Vogastapi, Vatnsleysuströnd, Reykjanes (63° 58′ 14.791″ N 22° 27′ 44.120″ W); (ii) a 2nd cal. year male (id. N. Fr-12–30) was found emaciated on 3 July 2012 in Önundarfjörður, Westfjords (65° 59′ 09.8″ N 23° 22′ 23.8″ W). The carcasses were frozen as a whole and stored in a freezer until dissection. Combined, a total of six adult and ten juvenile *Strigea* spp. were found in the birds’ intestines. The worms were recovered from the intestines under a stereomicroscope, washed in saline, rinsed and fixed in 70% ethanol for further analyses. We followed the concept of Pleijel et al. (2008) and cut a small piece of the posterior part of two adult specimens (hologenophores) for DNA sequence analyses; one well-preserved, gravid adult was kept as a paragenophore.

### Morphological examination

The material of the recovered worms was identified with aid of Dubois (1968) and the key of Niewiadomska (2002). The three vouchers (two hologenophores and the paragenophore, see above) were stained in iron acetocarmine, dehydrated in ethanol, cleared in clove oil and mounted in Canada balsam. Detailed morphological examination was carried out with aid of a light microscope Olympus BX51; drawings were made with aid of a drawing attachment. Measurements were taken from microphotographs of total mounts in Canada balsam with aid of ImageJ image analysis software (Schneider et al. 2012). All measurements are given in micrometres as the range.

For the description, we used the terminology of Niewiadomska (2002); for anterior and posterior parts of body, we used the terms ‘prosoma’ and ‘opisthosoma’ proposed by Achatz et al. (2021) for Diplostomoidea.

The voucher material of three specimens of *S. falconis* is deposited in the Icelandic Institute for Natural History (IINH), Urriðaholtsstræti 6, 210 Garðabær, Iceland.

### DNA processing and phylogenetic analyses

A small piece of the posterior part of two adult specimens (hologenophores ex birds Fr-12–30 and Fr-12–39) was used for the molecular analysis (Table 1). After removing ethanol from the samples, DNA was extracted using the QIAamp DNA Mini Kit (Qiagen, Germany) following manufacturer’s instructions. During the first step (tissue lysis), parasites were crushed one by one using a piston pellet (Treff, Switzerland). Polymerase chain reactions (PCR) of the D2 domain of the 28S subunit of rDNA were performed under conditions described by Jouet et al. (2009) with primers C2′B (5′-GAA AAG TAC TTT GRA RAG AGA-3′) and D2 (5′-TCC GTG TTT CAA GAC GGG-3′) (Mollaret et al. 1997). Two domains of the mitochondrial DNA were amplified and sequenced using a couple of primers: (i) forward NDJ11 (5′-AGA TTC GTA AGG GGC CTA ATA-3′) and reverse NDJ2a (5′-CTT CAG CCT CAG CAT AAT-3′) for the cytochrome c oxidase subunit I (*cox*1) according to Kostadinova et al. (2003); (ii) and JB3 (5′-TTT TTT GGG CAT CCT GAG GTT TAT-3′) and JB4.5 (5′-TAA AGA AAG AAC ATA ATG AAA ATG-3′) for the NADH dehydrogenase subunit 1 (ND1) according to Bowles et al. (1995). PCR products were directly sequenced in both directions with the primers used for DNA amplification (GenoScreen, France). The sequences obtained from this study are deposited in GenBank under the accession numbers PP218148–PP218149, PP229533–PP229534 and PP239093–PP239094 (Table 1).

**Table 1:** Isolates of *Strigea falconis* from the present study used for molecular analyses

Taxa	Stage	Host	Ref	Origin	Accession Numbers		
					D2	ND1	Cox1
STR2	Adult	*Falco rusticolus*	Fr-12–39	Iceland	PP229533	PP239093	PP218148
STR3	Adult	*Falco rusticolus*	Fr-12–30	Iceland	PP229534	PP239094	PP218149

Sequences were aligned using ClustalW that is included in MEGA version 11 software (Tamura et al. 2021) and then checked by eye. Sequences obtained were compared with sequences of Strigeidae previously identified from different developmental stages available in GenBank Database (*Apatemon* spp.: 158 sequences, *Apharyngostrigea* spp.: 57 sequences, *Australapatemon* spp.: 313 sequences, *Cardiocephaloides* spp.: 65 sequences, *Cotylurus* spp.: 286 sequences, *Ichthyocotylurus* spp.: 26 sequences, *Nematostrigea* spp.: 6 sequences, *Parastrigea* spp.: 77 sequences, *Strigea* spp.: 69 sequences and Strigeidae sp.: 61 sequences). The D2 domain of the rDNA (556 bp) and the partial ND1 (395 bp) and *cox*1 (236 bp) domains of the mDNA were used for molecular comparisons and tree constructions. Phylogenetic trees were constructed based on previous analyses (Heneberg et al. 2018; López-Jiménez et al. 2023) and new taxa available in GenBank (Table 2), using the neighbour joining (NJ), the maximum likelihood (ML) and minimum evolution (ME) methods, using the MEGA version 11 software. Bayesian inference (BI) analysis was conducted using MrBayes v. 3.2.6 (Huelsenbeck and Ronquist 2001; Dereeper et al. 2008, 2010). Markov chain Monte Carlo (MCMC) chains were run for 10,000,000 generations, and a sampling frequency of 100 generations resulted in 100,000 generations being saved. A burn‐in setting of 25% was used. The most appropriate nucleotide substitution model was determined (HKY + G for D2 and ND1; HKY + G + I for *cox*1). For NJ, ML and ME analyses, gaps were treated as missing data and internal node support was assessed by bootstrapping over 1000 replicates. *Tylodelphys aztecae* (Acc. N. MF398837), *Hysteromorpha triloba* (Acc. N. MH536511) and *H. triloba* (Acc. N. MF628069) were used as outgroup for D2, ND1 and *cox*1 domains, respectively. Visualisation of the trees and distance matrices for all datasets were calculated in MEGA ver. 11 (Tamura et al. 2021).

**Table 2:** Sequences of Strigeidae used for phylogenetic analyses

Genus/species	Author	Host	Stage*	Country	D2 (28S)	ND1	Cox1
*Strigea*							
*S. falconis*	Heneberg et al. (2016)		A	Czech Republic		KT074969–970	
	Heneberg et al. (2018)	*Aquila pomarina*	A	Czech Republic		MF628024	
		*Buteo buteo*	A	Czech Republic		MF628025	
		*Accipiter nisus*	A	Czech Republic		MF628026	MF628043
		*Aquila heliaca*	A	Czech Republic		MF628027	MF628044
		*Buteo buteo*	A	Czech Republic		MF628028	MF628045
		*Circus aeruginosus*	A	Czech Republic		MF628029	MF628046
		*Falco subbuteo*	A	Czech Republic			MF628048
		*Circus aeruginosus*	A	Czech Republic			MF628047
		*Aquila pomarina*	A	Czech Republic			MF628042
*S. magnirostris*	López-Jiménez et al. (2023)	*Rupornis magnirostris*	A	Mexico	OQ647909		
					OQ647910–914		OQ648142–146
					OQ647917		OQ648135
		*Accipiter cooperii*	A	Mexico	OQ647915–916		OQ648140–141
							OQ648147
		*Rupornis magnirostris*	A	Mexico	OQ647918–922		OQ648136–139
							OQ648148–150
*S. robusta* (syn. *P. robusta*)	Sinsch et al. (2019)	*Lissotriton vulgaris*	Mt	Germany			MF537209–212
	Svinin et al. (2020)	*Planorbarius corneus*	C	Russia	MT075841–842		
		*Pelophylax esculentus*	Mt	Russia	MK585229–230		
	Heneberg et al. (2018)	*Anas platyrhynchos*	A	Poland			MF628067
	Svinin et al. (2022)	*Planorbis planorbis*	C	Russia			OM943856–858
*S. sphaerula*	Heneberg et al. (2018)	*Acrocephalus arundinaceus*	A	Czech Republic			MF628054
*S. strigis*	Svinin et al. (2023)	*Planorbis planorbis*	C	Russia	OP714364		OP715857
	Heneberg et al. (2018)	*Asio otus*	A	Czech Republic		MF628030–032	MF628049–051
*S. vandenbrokae*	Heneberg et al. (2018)	*Pernis apivorus*	A	Czech Republic		MF628033	MF628052–053
*Strigea* sp.	Hernández-Mena et al. (2017)	*Caracara cheriway*	A	Mexico	MF398343		MF398319
	Patrelle et al. (2015)	*Pelophylax* sp.	Ms	France	KT362372–373		
*Strigea macrobursa*	López-Jiménez et al. (2023)	*Buteogallus urubitinga*	A	Mexico	OQ647923–930		OQ648128–133
		*Buteogallus anthracinus*	A	Mexico	OQ647931		OQ648134
*Parastrigea*							
*P. plataleae*	Hernández-Mena et al. (2017)	*Platalea ajaja*	A	Mexico	MF398346		
*P. cincta*	Hernández-Mena et al. (2017)	*Eudocimus albus*	A	Mexico	MF398347		
*P. diovadena*	Hernández-Mena et al. (2017)	*Eudocimus albus*	A	Mexico	MF398348		
*P. flexilis*	Heneberg et al. (2018)	*Circus aeruginosus*	A	Czech Republic		MF628040	MF628065
*Nematostrigea*							
*N. serpens*	Lebedeva and Yakovleva (2016)	*Pandion haliaetus*	A	Russia	KF434762		
*Ichthyocotylurus*							
*I. erraticus*	Olson et al. (2003)	*Coregonus autumnalis*	A	Northern Ireland	AY222172		
	Anandan et al. (2003) (DS)*	*Perca fluviatilis*	Mt	England		AY386170	
*I. pileatus*	Heneberg et al. (2018)	*Sterna hirundo*	A	Czech Republic			MF628068
*Cotylurus*							
*C. syrius*	Pyrka et al. (2021)	*Cygnus olor*	A	Poland	MW244647		
	Heneberg et al. (2018)	*Cygnus olor*	A	Czech Republic		MF628034	MF628056
						MF628036	MF628057–059
*C. stigeoides*	Pyrka et al. (2021)	*Anas platyrhynchos*	A	Poland	MW244640		
*C. raabei*	Pyrka et al. (2021)	*Anas platyrhynchos*	A	Poland	MW244649		
*C. hebraicus*	Pyrka et al. (2021)	*Fulica atra*	A	Poland	MW244638		
*C. cornutus*	Soldánová et al. (2017)	*Radix balthica*	Mt	Norway	KY513180		
	Heneberg et al. (2018)	*Anas crecca*	A	Czech Republic			MF628064
*C. marcoglieseli*	Locke et al. (2018)	*Lophodytes cucullatus*	A	Canada		MH536509	
*Cardiocephaloides*							
*C. physalis*	Vermaak et al. (2021)	*Spheniscus demersus*	A	South Africa	MW370425		
		*Spheniscus magellanicus*	A	Chile			MN817947
*C. ovicorpus*	Bennett et al. (2023)	*Tripterygiidae* sp.	Mt	New Zealand	OQ407749		
*C. medioconiger*	de Buron et al. (2023)	*Lobotes surinamensis*	Mt	USA	OP761874		
	Locke et al. (2018)	*Thalasseus maximus*	A	USA		MH536508	
*C. longicollis*	Olson et al. (2003)	*Chroicocephalus ridibundus*	A	Ukraine	AY222171		
*Australapatemon*							
*Au. niewiadomski*	Blasco-Costa et al. (2016)	*Anas platyrhynchos*	A	New Zealand	KT334165		KT334180
*Au. burti*	Hernández-Mena et al. (2017)	*Anas diazi*	A	Mexico	MF398342		
*Au. minor*	Heneberg et al. (2018)	*Anas platyrhynchos*	A	Czech Republic		MF628041	MF628066
*Apharyngostrigea*							
*Aph. pipientis*	Pulis et al. (2011)	*Nycticorax nycticorax*	A	USA	JF820597		
	Locke et al. (2021)(DS)*	*Botaurus lentiginosus*	A	Canada		NC059570	
	Locke et al. (2021)	*Botaurus lentiginosus*	A	Canada		MT679576	
*Aph. cornu*	Tkach (2001)				AF184264		
*Aph. brasiliana*	López-Jiménez et al. (2022)	*Cochlearius cochlearius*	A	Mexico	MZ614713		
*Apatemon*							
*A.* sp. *jamiesoni*	Blasco-Costa et al. (2016)	*Phalacrocorax punctatus*	A	New Zealand	KT334169		KT334182
Aff. *Apatemon* sp.	Coleman (2012) (DS)*	*Galaxiella pusilla*	Mt	Australia			JX051347
*A. gracilis*	Soldánová et al. (2017)	*Gasterosteus aculeatus*	Mt	Norway	KY513177		
*A. fuligulae*	Pyrka et al. (2021)	*Aythya ferina*	A	Poland	MW244636		
	Heneberg et al. (2018)	*Aythya ferina*	A	Czech Republic			MF628055
*A.* cf*. hypseleotris*	Rochat et al. (2020)	*Hypseleotris* sp.	Mt	Australia	MT603884		
*A. fuhrmanni*	Heneberg et al. (2018)	*Cygnus olor*	A	Czech Republic		MF628035	MF628060
						MF628038–039	MF628061–063
Strigeidae sp.	Hammoud et al. (2022)	*Bulinus tropicus*	C	Uganda		ON970217	
	Hammoud et al. (2022) (DS)*	*Bulinus tropicus*	C	Uganda		OQ574601–607	
	Iwaki et al. (2014) (DS)*	*Turdus naumanni*	Mt	Japan	LC011455		

^*^Abbreviations: (DS), direct submission, unpublished; A, adult stage; C, cercaria; Mt, metacercaria

## Results

### Morphological description of the voucher material

**Strigeidae Railliet, 1919**

***Strigea*** **Abildgaard, 1790**

***Strigea falconis*** **Szidat, 1928**

Host: *Falco rusticolus* L., 1758.

Locality: Önundarfjörður Westfjords Region (65° 59′ 09.8″ N 23° 22′ 23.8″ W); Vogastapi, Vatnsleysuströnd, Reykjanes Peninsula (N 63° 58′ 14.791″ W 22° 27′ 44.120″), Iceland.

Site in host: small intestine.

Infection rates: intensity—2 adult specimens and 10 juveniles per bird from 2011; 4 adult worms per bird from 2012.

Material: two hologenophores STR2 (Coll. No. IINH 301234, ex bird Fr-12–39, coll. 31.v. 2011) and STR3 (Coll. No. IINH 301235, ex bird Fr-12–30, coll. 3.vii. 2012), one paragenophore STR1 (Coll. No. IINH 301233, ex bird Fr-12–39, coll. 31.v. 2011).

Representative DNA sequences: Isolates STR2 and STR3 (see Table 1)—EA7510 ESCAPE-DJ-Reims, France.

#### ***Adult (***Fig. 1***)***

**Fig. 1:**
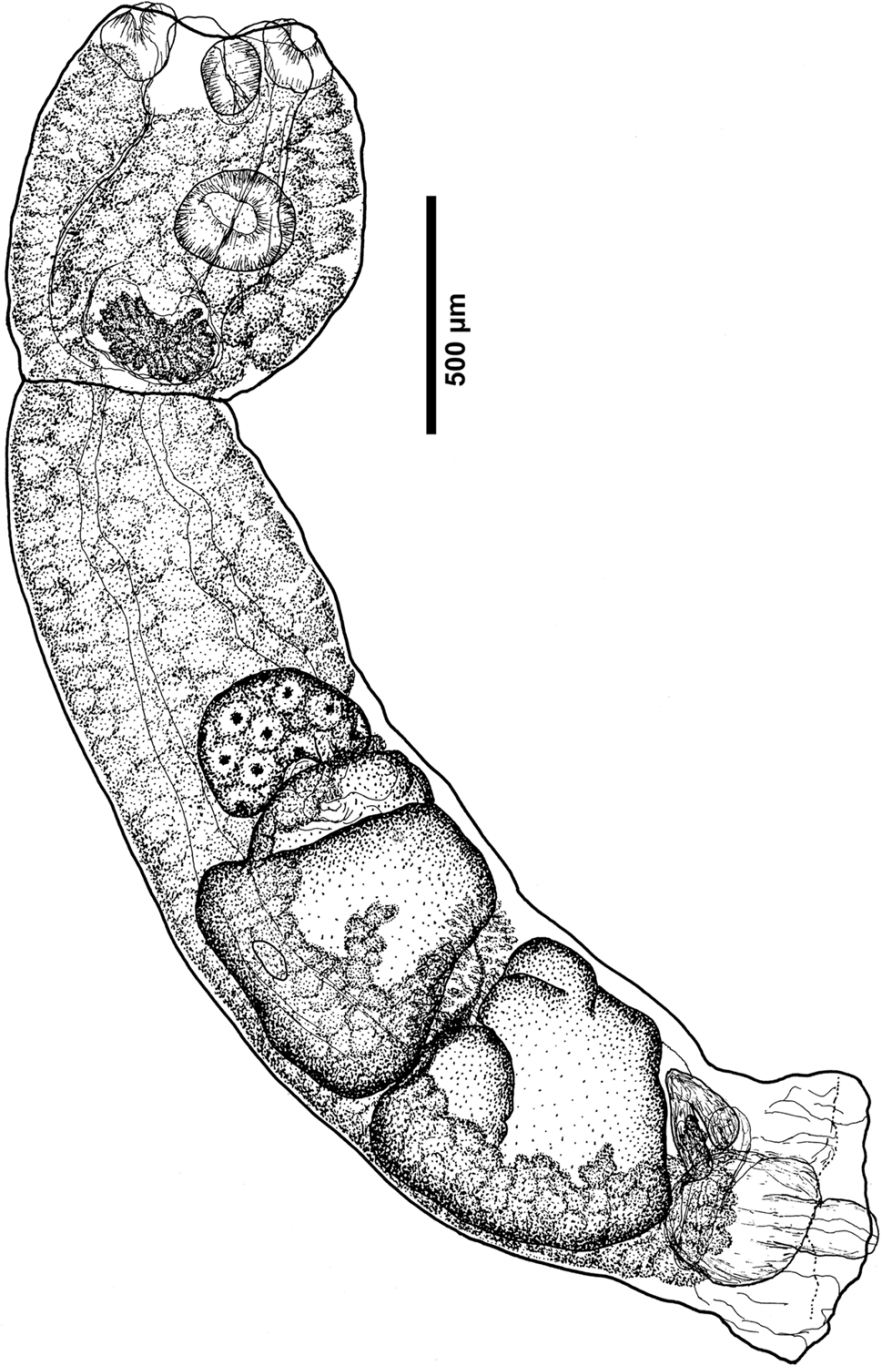
Paragenophore (STR1) of *Strigea falconis* ex *Falco rusticolus* (Fr-12–39), lateral view

[Description and measurements based on three specimens fixed in ethanol (not all specimens contributed a data point to all variables, e.g., total body length taken only from paragenophore).] Body distinctly bipartite, neck-region absent, slightly curved, 3313 long. Prosoma cup-shaped, with maximum width at its mid-level, well separated from opisthosoma, 766–903 × 666–827. Opisthosoma long, sub-cylindrical, elongate, with almost parallel margins, slightly curved, 2546 × 655–892, with slight constriction just anterior to copulatory bursa. Opisthosoma much longer than prosoma; opisthosoma/prosoma length ratio 1:3.3. Tegument smooth.

Oral sucker small, weakly muscular, elongate-oval, ventro-subterminal, 75–143 × 68–113. Ventral sucker weakly muscular, sub-spherical, at mid-level of prosoma; larger than oral sucker, 197–217 × 166–247; ventral sucker/oral sucker length ratio 1:1.52–2.62. Pseudosuckers present, of similar size as oral sucker. Prepharynx absent. Pharynx well-developed, feebly muscular, 74–123 × 61–90. Oesophagus, intestinal bifurcation and caeca in prosoma not observed. Caeca long, narrow, posterior extension not observed. Holdfast organ in prosoma, consisting of two large lobes, slightly protruding out of prosoma, with a deep slit. Proteolytic gland distinct, at base of prosoma, 166–262 × 192–287.

Testes two, large, tandem, lobed, post-ovarian, contiguous, in second and last third of opisthosoma; post-testicular region 434 long, 17% of opisthosoma length. Anterior testis square-shaped to transversely-oval, with three lobes turned dorsally, 313–635 × 565–824. Posterior testis slightly smaller, square-shaped or asymmetrical, with two lobes turned dorsally, 650 × 577–782. Seminal vesicle coiled, post-testicular, relatively small.

Ovary reniform, entire, or lobed, sub-median, pretesticular, contiguous with anterior testis; in second quarter of opisthosoma, 176–232 × 166–368. Vitellarium follicular, vitelline follicles numerous, small, present in prosoma and opisthosoma. In prosoma, vitellarium extending up to its three quarters or more. In opisthosoma, vitelline follicles confluent anteriorly, filling its full width up to level of ovary, at level of testes forming a ventral field expanding to half-width of body, in post-testicular region confluent, extending ventrally to copulatory bursa. Vitelline reservoir conspicuous, between testes. Mehlis’ gland diffuse, dorsal, between testes. Uterus extending well in front of ovary (first quarter of opisthosoma), continuing on ventral side towards posterior end. Eggs numerous, 83–111 × 55–66. Copulatory bursa large, 454 × 618, becoming wider towards posterior extremity, opening terminally, occupying 18% of opisthosoma length. Genital cone well delimited from parenchyma, with muscular ring (Ringnapf), just post-testicular, 452 × 288. Excretory vesicle and excretory pore not observed.

### Remarks

The present material agrees well with the diagnosis of the genus *Strigea* Abildgaard, 1790 by Niewiadomska (2002) in the presence of a distinctly bipartite body, with vitelline follicles evenly distributed in both prosoma and opisthosoma, in prosoma extending into body wall and holdfast organ, well-developed pharynx, multilobed testes and large copulatory bursa with genital cone well delimited from parenchyma and with a muscular ring.

The morphology of our present material agrees well with the description of *Strigea falconis* by Dubois (1938, 1968) in the presence of a cup-shaped prosoma with the holdfast organ slightly protruding from its opening, well-developed suckers and pharynx, large, lobed testes, vitelline follicles evenly distributed in both parts of body, in prosoma never covering pharynx and oral sucker and terminating near the copulatory bursa in opisthosoma, and well-pronounced copulatory bursa with a well-delimited genital cone. Our morphometric data fall within the dimensions provided by Dubois (1938, 1968), only the oral sucker and pharynx show lower minima, while testes are wider. When comparing our dimensions to those of Heneberg et al. (2018), who provided measurements of 30 individuals ex *Buteo buteo* linked to sequences, our material overlaps as well; however, it exhibits lower minima for prosoma, suckers, pharynx, anterior ovary width and anterior testis length, while on the other side, the sucker length ratio shows slightly higher maxima (1:1.52–2.62 vs. 1:1.3–2.3) (see also Supplementary Information, Table S1). Our material of *S. falconis* can be well distinguished from *S. falconis brasiliana* Szidat, 1929, considered a subspecies by Dubois (1968). Our specimens of *S. falconis* differ in size relation of the genital cone to the ovary (genital cone of similar size as ovary vs. genital cone larger than ovary), in larger eggs (83–111 × 55–66 vs. 67–91 × 42–55), and in geographical distribution, as *S. falconis brasiliana* is restricted to Cuba and South America (see Dubois 1968; Drago et al. 2015; and Table S1). Based on phylogenetic analyses, *S. falconis* is most close to *S. magnirostris* López-Jiménez et al., 2023 and *S. macrobursa* (Drago & Lunaschi, 2011) from Mexico and South America. Our material of *S. falconis* differs from *S. magnirostris* in absence of papillae on oral sucker, absence of neck region in opisthosoma, tegument being devoid of spines, in the opisthosoma to prosoma ratio (3.3 vs. 3.3–5.3) and in possessing larger testes. From *S. macrobursa*, our material differs in larger body (3313 vs. 957–2880), lobed testes (vs. not lobed), presence of a muscular ring (vs. muscular ring absent), absence of tegumental spines and very shallow genital atrium (vs. very deep). From *S. macropharynx* described from a gyrfalcon in Alaska by Dubois and Rausch (1965), our material differs in a larger body (3313 vs. 790–2110) and larger prosoma (766–903 × 666–827 vs. 450–700 × 320–480), in the presence of a much smaller pharynx (74–123 × 61–90 vs. 135–165 × 110–145) being substantially smaller than oral sucker (vs. pharynx larger than oral sucker), and a short, inconspicuous hermaphroditic duct (vs. long, muscular). From the North American *S. macroconophora* Dubois & Rausch, 1950 ex *Buteo jamaicensis borealis* with similarly distributed vitelline follicles (see Dubois and Rausch 1965), our material differs in possessing larger testes, a smaller genital cone (452 × 288 vs. 340–660 × 405–510) and a smaller copulatory bursa (taking c. 1/5 of opisthosoma length vs. taking 1/3 of opisthosoma length) and a wider extent of vitelline follicles in prosoma (extending up to its three quarters or more vs. extending to its half) (see also Table S1).

### Molecular identification and phylogenetic analyses

**Fig. 2:**
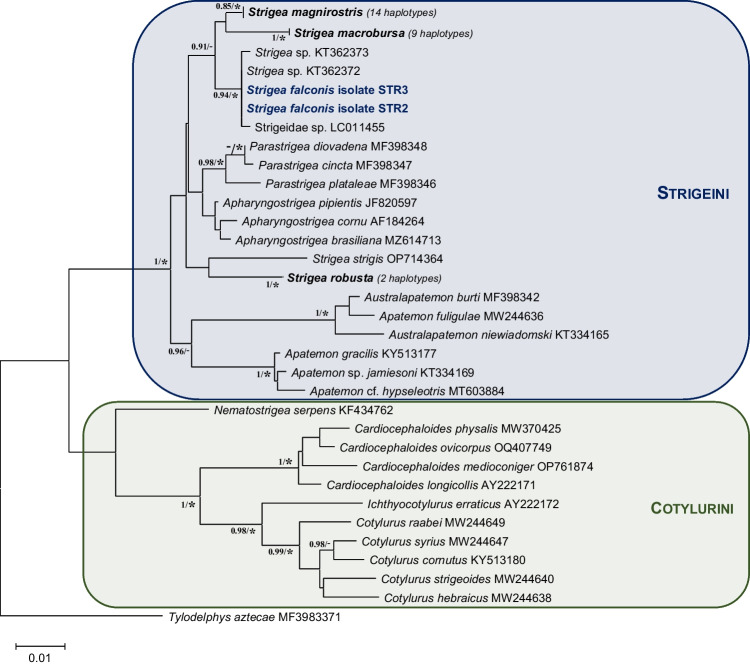
Phylogenetic tree based on the D2 domain of rDNA of sequences of Strigeidae constructed using the maximum likelihood (ML) method. The scale shows the number of nucleotide substitutions per site between DNA sequences. Nodal support from Bayesian inference (BI) and neighbour joining (NJ), maximum likelihood and Minimum evolution (ME) analyses indicated as BI/(NJ-ML-ME), with values > 0.90 (BI) and ‘*’ for bootstrap values higher than 85% in ML, NJ and ME

The Bayesian inference and phylogenetic analyses (NJ, ML and ME) of the ribosomal (D2) and mitochondrial (ND1 and *cox*1) are congruent with previous studies and confirmed the separation of the Strigeidae in two major clades, corresponding to the two tribes of Strigeinae Railliet, 1919, i.e. Strigeini Dubois, 1936 and Cotylurini Dubois, 1936 (Fig. 2).

Molecular analyses and comparisons of the D2 domain of the large ribosomal subunit (rDNA) were conducted based on 55 sequences available in GenBank from 9 genera and 30 species or lineages of Strigeidae and sequences obtained in the present study. No variation was observed between our sequences isolated from gyrfalcons in Iceland and the sequence of *Strigea* sp. (Acc. N. KT362372) of a mesocercaria isolated from *Pelophylax* sp. in France, and only one variation (i.e. a homology of 99.4%) with the sequence of *Strigea* sp*.* (Acc. N. KT362373) also of a mesocercaria from *Pelophylax* sp. in France and the sequence of Strigeidae sp. (Acc. N. LC011455) of a metacercaria from *Turdus naumanni* in Japan was observed. The phylogenetic analysis of this domain (Fig. 2) shows that the genus *Strigea* is split into two separate clades (confirmed by intraspecific and interspecific variations, see Tables S2 and S3): (i) the first clade includes the type-species *Strigea strigis*, as well as the European species *Strigea robusta* (syn. *Parastrigea robusta*); (ii) the second clade contains the South American species *Strigea magnirostris*, *Strigea macrobursa* (syn. *P. macrobursa*), the two haplotypes of *Strigea* sp. and Strigeidae sp. and sequences from this study. These two clades of *Strigea* spp. are separated by another clade comprising the genera *Parastrigea* Szidat, 1928 and *Apharyngostrigea* Ciurea, 1927 (see Fig. 2).

Molecular analyses and comparisons of the ND1 and partial *cox*1 domains of the mDNA were used at the generic and specific level. For the ND1 (395 bp, 35 sequences), intraspecific variations (4 to 6 variations, i.e. homology of 99.2%) between haplotypes STR2 and STR3 and sequences of *Strigea falconis* isolated from *Accipiter nisus*, *Aquila pomarina*, *Aquila heliaca*, *Buteo buteo* and *Circus aeruginosus* from the Czech Republic (Acc. Ns. MF628024–MF628029 and KT074969–KT074970), and interspecific variations (73 to 88 variations) with other species of *Strigea* confirmed the affiliation of our adults from gyrfalcons to the European *S. falconis*.

Molecular and phylogenetic analyses of the partial *cox*1 (236 bp, 64 sequences) show the presence of two clades within the *Strigea* spp.: (i) a clade comprising the type-species *Strigea strigis*, as well as the species *S. robusta*, *S. macrobursa*, *S. sphaerula* and *S. vandenbrokae*; (ii) a second clade comprising the species *S. magnirostris* and *S. falconis*. Intraspecific variations (6 variations, 99.9% of homology) confirmed that the haplotypes of adults isolated from gyrfalcon in Iceland belong to the species *Strigea falconis* (see Table S4). Interspecific variability with other species of *Strigea* spp. varies from 12.9% (29 variations—e.g. *S. falconis *vs.* S. magnirostris*) to 14.9% (50 variations—e.g. *S. falconis *vs.*﻿ S. robusta*) (see Table S5).

**Fig. 3:**
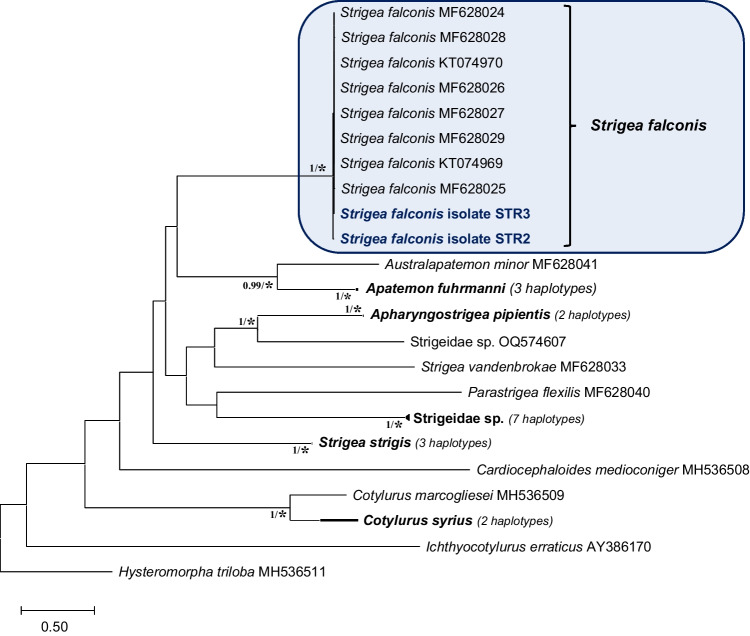
Phylogenetic tree based on the ND1 domain of mDNA of sequences of Strigeidae constructed using the maximum likelihood (ML) method. The scale shows the number of nucleotide substitutions per site between DNA sequences. Nodal support from Bayesian inference (BI) and neighbour joining (NJ), maximum likelihood and minimum evolution (ME) analyses indicated as BI/(NJ-ML-ME), with values > 0.98 (BI) and ‘*’ for bootstrap values higher than 95% in ML, NJ and ME

**Fig. 4:**
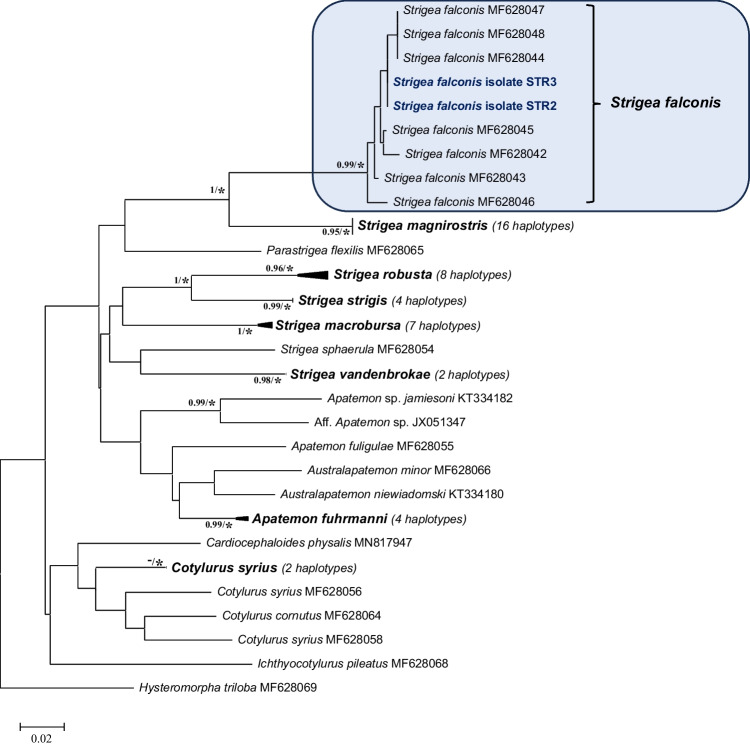
Phylogenetic tree based on the *cox*1 domain of mDNA of sequences of Strigeidae constructed using the maximum likelihood (ML) method. The scale shows the number of nucleotide substitutions per site between DNA sequences. Nodal support from Bayesian inference (BI) and neighbour joining (NJ), maximum likelihood and minimum evolution (ME) analyses indicated as BI/(NJ-ML-ME), with values > 0.95 (BI) and ‘*’ for bootstrap values higher than 95% in ML, NJ and ME

For both ND1 and partial *cox*1 domains, all of the well-known species represent distinct taxa well supported by BI and bootstrap values (see Figs. 3 and 4).

## Discussion

In the present study, we provide the first record of *S. falconis* from Iceland, and at the same time, the first record of *S. falconis* from a gyrfalcon host supported by molecular genetic characterisation combined with morphology. From Iceland, there were reports on *Strigea* sp. by Christensen (2013) and Christensen et al. (2015). The species-level identification of their record is accomplished in the present study, supporting the wide geographical distribution of *S. falconis* and proving that it extends to the sub-Arctic.

The first record of *S. falconis* from *F. rusticolus islandus* was provided by Baylis (1939) from Argyllshire, Scotland; however, without any further details, therefore we can only speculate about the origin of the Icelandic gyrfalcon in Scotland. Apart from the finding of Baylis (1939), there is only one more record of *S. falconis* from gyrfalcons from a kennel in Tuva, Russia, in South Siberia (Davydova et al. 2016); however, this identification should be viewed with caution, as it is based on eggs only and is not supported by molecular genetic data. The only other strigeid from a gyrfalcon is *S. macropharynx* described by Dubois and Rausch (1965) from Alaska, USA. This scarcity of trematode records from gyrfalcon is most probably because of the birds’ restricted geographical distribution (Arctic, sub-Arctic) and their trophic specialisation on rock and willow ptarmigan, which are not the permissive intermediate hosts for transmission of metacercariae to gyrfalcons. Therefore, we assume that gyrfalcons are rather uncommon hosts of trematodes. Although gyrfalcons prey upon a wide variety of birds other than ptarmigan, it seems that the trematodes so far recorded in Iceland could have been ingested rather accidentally with prey (*Cryptocotyle* spp., *L. propinqua*, *M. pygmaeus* and *P. elegans*; see Christensen 2013; Christensen et al. 2015).

The present case of *S. falconis* in Iceland is probably the most northern record of this species, and it is beyond doubt unusual. Based on the information we have on the life cycle of *S. falconis*, it is impossible to be completed in Iceland. The Icelandic gyrfalcon population is fully sedentary, i.e. not migrating southwards to Europe for wintering (Nielsen and Cade 1990a), where it could get a trematode infection as it happens, e.g. to waterfowl acquiring bird schistosomes in wintering places (Kolářová et al. 2006; 2013; Jouet et al. 2015). There are no appropriate snail intermediate hosts present in Iceland, although there is a recent single record of *P. planorbis* from central Iceland (Academy of Natural Sciences (ANS) 2024), which is unusual as this snail species is not known to expand to higher latitudes (Glöer 2019). And even though there are small planorbid snails (*Gyraulus parvus* (Say) and *Bathyomphalus contortus* (L.)) (Faltýnková et al., 2023; own observation), which potentially could act as alternative first intermediate hosts, other organisms serving as second intermediate hosts (amphibians and reptiles) are missing completely. The only plausible explanation is that the Icelandic gyrfalcons acquired their infection by preying upon migratory birds (infected with metacercariae) arriving from Europe where they got infected during wintering by ingesting amphibians or reptiles carrying mesocercariae of *S. falconis*.

As mentioned above, the main diet of gyrfalcons is ptarmigan, but this type of prey can be supplemented by other birds, such as waterfowl, waders, shorebirds, gulls, auks, passerines and even other raptors in summer and winter, of which many are migratory (Nielsen 2003, 2011; Nielsen and Cade 1990b, Table S6). This spectrum might overlap with the wide array of bird species recorded as hosts of metacercariae of *S. falconis* (previously also reported under the name *Tetracotyle ardeae* Mataré, 1910) including Ciconiiformes, Passeriformes, Podicipediformes, Ralliformes, Pelecaniformes, Anserifores, Charadriiformes, Falconiformes, Coraciiformes, Galliformes, Columbiformes, Strigiformes and Piciformes (Sudarikov 1959, 1984; Bykhovskaya-Pavlovskaya 1962; Dubois 1968). Several species of these bird families were recorded as prey items of gyrfalcons in Iceland, indicating mainly species of Anseriformes and Charadriiformes as the most likely candidates (see Table S6). As the location of the metacercariae in birds, the subcutaneous tissue, the area near oesophagus or trachea, body cavity and adipose tissue was reported (Odening 1967; Dubois 1968; Krone and Streich 2000; Syrota et al. 2021). In more recent time, in Germany, *Buteo buteo* was reported to host metacercariae of *S. falconis* by Krone and Streich (2000), while in Ukraine, *Ardea purpurea* and *Nycticorax nycticorax* were recorded (Syrota et al. 2021); in Korea, *Egretta alba modesta* Gray was recorded by Ryang et al. (1991). Certainly, the reports are scarce now because it is nearly impossible to obtain birds for parasitological examination, but the few reports document the presence *S. falconis* in the ecosystems. On the other hand, the fairly wide host spectrum recorded in the older literature casts some doubt if only *S. falconis* was involved, particularly because metacercariae are the life cycle stage with least characters for identification. The birds act as third intermediate and most likely as paratenic hosts, accumulating metacercariae over time (long-time survival of metacercariae in their hosts is known) and ensuring their dispersion. As Iceland lies on the East Atlantic bird flyway, we can assume that a transfer of metacercariae by migratory birds is highly likely. The western part of Iceland (the Reykjanes Peninsula and the Westfjords) is used by a wide array of bird species for breeding, and some species stop in this region while migrating to Greenland (Wilson 1981; Garðarsson 1999; Doyle et al. 2021).

The taxonomy of the Strigeinae is still controversial, due to the absence of easily identifiable morphological characters, or variations depending on the stage or maturity of the parasites, and the hosts from which they have been isolated, leading to misidentifications in the past (Heneberg et al. 2018). The recent use of an integrative approach combining morphology, molecular biology and the study of parasites and their hosts is essential and has made it possible to confirm or place species in synonymy and to understand the taxonomy within several genera of this family (Blasco-Costa et al. 2016; Achatz et al. 2020; Locke et al. 2021; Pyrka et al. 2021; Sokolov et al. 2021). Regarding the genus *Strigea*, López-Jiménez et al. (2023) analysed the South American species based on different nuclear and mitochondrial domains. Heneberg et al. (2018) showed the separation of the European species into three clades (*Strigea *sensu stricto, *Amphistoma* and *Cryptostrigea*), proved the well-defined status of *S. falconis* and assumed that it might belong to a separate genus (*Cryptostrigea*). The new species described since then and the results obtained in this study confirm this hypothesis, and another recently described species, *Strigea magnirostris*, falls in the *Cryptostrigea* clade. However, there is still not enough evidence to put the transfer to *Cryptostrigea* into effect because more molecular data for other species and genera are needed to reinforce this hypothesis. Records supported by molecular genetic data are still scarce for *S. falconis*, apart from Heneberg et al. (2018), the only other finding is that of mesocercariae ex* Natrix natrix* from Poland by Bełcik et al. (2022), whose molecular data concern only the small subunit of ribosomal RNA gene, making a comparison with our data impossible. Moreover, concerning haplotypes of the adults isolated from the gyrfalcons in Iceland, our material corresponds morphologically to those found in Central Europe in *Buteo buteo* of which Heneberg et al. (2018) provided DNA sequences. Our analyses also show a high degree of similarity in D2 between our haplotypes of *S. falconis* and the sequences of mesocercariae (*Strigea* sp.) isolated from frogs in France (Patrelle et al. 2015). This could indicate the circulation of *S. falconis* in wetlands in France, with frogs being the source of infection for aquatic birds. Given the conserved nature of 28S ribosomal DNA, and the absence of sequences for other mitochondrial domains for these taxa, a comparison with other markers is necessary to confirm their identity. For the D2 (28S), our haplotypes are also close to the sequence of a metacercaria (Strigeidae sp.) from *Turdus naumanni* in Japan (Iwaki et al. 2014, unpublished). Unfortunately, the lack of sequences for other genetic domains and of additional data on this taxon means that we are unable to identify whether it could be the same species. Finally, for a better phylogenetic resolution, more genetic data from other continents than Europe are needed (only seven species are available out of 47 described), particularly from South America. Obtaining sequences especially of *Strigea falconis var. brasiliana* is of interest because this sub-species, which has been frequently found, shows substantial morphological differences (see ‘Remarks’) to the Holarctic species, suggesting that it could be a well-defined, separate species.

*Strigea falconis* illustrates that a complex life cycle with multiple intermediate hosts (including paratenic hosts) can be advantageous for a parasite (see Poulin 2007; Parker et al. 2015; Benesh et al. 2021), particularly in combination with low host specificity, and can lead to its wide dispersion. This is indicated by the large-scale distribution of *S. falconis* and its common occurrence in birds of prey. At the same time, this case study shows one of the pitfalls of such a complex life cycle when a parasite is introduced to a new area with no suitable intermediate hosts. Thus, gyrfalcons in Iceland are apparently dead-end hosts for *S. falconis*.

### Supplementary Information

Below is the link to the electronic supplementary material.

Supplementary file1 (266 KB)

## Data Availability

Data are available from the authors upon reasonable request; sequences are made available via GenBank.
