# Supplementary material for: First species record of Strigea falconis Szidat, 1928 (Trematoda, Strigeidae) from gyrfalcon Falco rusticolus in Iceland—pros and cons of a complex life cycle
Source: Parasitol Res. 2024 Mar 4;123(3):147. doi: 10.1007/s00436-024-08161-w (PMC10909778; doi:10.1007/s00436-024-08161-w)
Supplement: Supplementary file 1 — Supplementary file1 (266 KB) [file 436_2024_8161_MOESM1_ESM.pdf]

Supplementary Files

Parasitology Research

First species record of *Strigea falconis* Szidat, 1928 (Trematoda, Strigeidae) from gyrfalcon *Falco rusticolus* in Iceland – pros and cons of a complex life-cycle

Anna Faltýnková[1], Damien Jouet[2], Ólafur Karl Nielsen[3], Karl Skírnisson[4]

[1]Department of Forest Ecology, Faculty of Forestry and Wood Technology, Mendel University in Brno, Zemědělská 3, Brno, 613 00, Czech Republic
[2]ESCAPE UR7510, USC ANSES PETARD, Faculty of Pharmacy, University of Reims Champagne-Ardenne, 51 rue Cognacq-Jay, 51096, Reims Cedex, France
[3]Icelandic Institute of Natural History, Garðabær, Iceland
[4]Laboratory of Parasitology, Institute for Experimental Pathology, Keldur, University of Iceland, IS-112 Reykjavík, Iceland

Correspondence
Anna Faltýnková, e-mail: faltyn.anna@gmail.com

Table S1 Comparative dimensions for *Strigea falconis* and related species

**Species**	*Strigea falconis*		*Strigea falconis braziliana*			*Strigea macrobursa*	*Strigea macroconophora*	*Strigea macropharynx*	*Strigea magnirostris*
**Location**		Czech Republic	Brazil, Cuba	Argentina	Argentina	Mexico	USA	Alaska, USA	Mexico
**Source**	Dubois (1938, 1968)	Heneberg et al. (2018)	Dubois (1968)	Lunaschi & Drago (2006)	Drago et al. (2018)	López-Jiménez et al. (2023)	Dubois and Rausch (1950), Dubois (1968)	Dubois and Rausch (1965), Dubois (1968)	López-Jiménez et al. (2023)
**Hosts**	*Accipiter*, *Buteo*, *Circus*	*Buteo buteo*	Accipitridae, Falconidae	*Rupornis* (*Buteo*) *magnirostris*	*Circus buffoni*	*Buteogallus urubitinga*	*Buteo jamaicensis borealis*, *Buteo platypterus*	*Falco rusticolus obsoletus*	*Rupornis* (*Buteo*) *magnirostris*
TBL	1830–5500	2714–4573 (3841)	up to 2500	1305–1392	2057–3285	957–2880	up to 3100	790–2110 (1980)	3030–4430
PSL	600–1500	857–1429 (1099)	380–590	319–415	677–1000	344–775	600–810	450–700 (590)	562–872
PSW	570–1500	714–1429 (1021)	420–700	314–367	740–985	238–562	730–1050	320–480 (400)	400–690
OPL	1230–3920	1714–3430 (2743)	1110–1830	890–1073	1371–2286	609–2184	1410–2340	1270–1540 (1390)	2440–3591
OPW	450–1560	629–1143 (863)	340–580	362–435	648–1000	256–759	750–900	380–600 (450)	352–632
OSL	95–200	116–174 (146)	100–125	76	68–126	64–95	100–153	100–140	77–109
OSW	–	128–192 (156)	85–115	55	89–169	57–86	85–120	105–140	80–115
VSL	180–315	203–290 (255)	160–235	152–162	101–212.5	74–106	190–200	195–245	150–240
VSW	115–315	203–290 (256)	140–200	71–105	121–266	55–98	140–160	150–220	124–188
PHL	80–160	104–192 (142)	73–95	74	75–140	33–66	100–119 (diam.)	135–165	65–103
PHW	–	104–128 (117)	70–95	48	58–124	31–59	–	110–145	64–90
PGL	140–200	–	105–130	–	109–193	75	–	150–210	182–225
PGW	200–300	–	120–190	–	169–270	43	–	70–105	83–119
OVL	135–310	174–368 (272)	110–200	59–68	179–280	52–188	130–190	125–180	139–190
OVW	170–380	230–740 (340)	175–300	101–107	203–493	72–193	250–270	100–135	126–214
ATL	300–900	368–644 (453)	235–360	169–227	198–541	95–281	370–600	200–250	272–476
ATW	235–690	460–1043 (636)	235–410	174–190	261–967	132–449	430–600	250–380	261–496
PTL	315–900	414–745 (551)	275–370	197–217	169–580	137–392	360–510	200–310	346–497
PTW	245–700	506–1043 (668)	235–420	179–241	285–880	155–474	440–595	270–390	280–512
CBL	–	–	–	183–241	295–561	163–681	–	–	247–531

CBW	–	–	–	215–226	257–493	246–616	–	–	468–794
GA	–	–	–	–	–	–	225–405	135–170	–
GCL	170–450 (260)	322–598 (480)	240–350	128–167	174–241.5	89–255	340–660	85–120 (diam.)	193–361
GCW	–	368–736 (526)	220–310	129–143	203–270	137–230	405–510	–	380–637
EL	75–110	99–110 (107)	67–91	82–88	93–117	72–117	90–99	105	71–105
EW	45–	64–75 (71)	42–55	48–52	57–74	45–67	63–70	60	40–65
OPL/PSL	1.60–3.44	1.7–3.3 (2.5)	1.9–3.6	2.1–3.4	1.7–2.4	1.5–3.1	2.9–3.1 (2.3–2.6)	–	3.3–5.3
VSL/OSL	–	1.3–2.3 (1.7)	–	–	–	–	–	–	–
VSW/OSW	–	1.3–2.0 (1.6)	1.6–1.7*	1.9	1.1–2 (1.5).	–	1.3–1.6*	–	1.45–2.42
OSL/PHL	–	–	0.8*	1.1 (0.9*)	–	–	0.99–1.4*	–	0.84–1.10

Abbreviations: TBL, total body length; PSL, prosoma length; PSW, prosoma width; OPL, opisthosoma length; OPW, opisthosoma width; OSL, oral sucker length; OSW, oral sucker width; VSL, ventral sucker length; VSW, ventral sucker width; PHL, pharynx length; PHW, pharynx width; PGL, proteolytic gland length; PGW, proteolytic gland width; OVL, ovary length; OVW, ovary width; ATL, anterior testis length; ATW, anterior testis width; PTL, posterior testis length; PTW, posterior testis width; CBL, copulatory bursa length; CBW, copulatory bursa width; GA, genital atrium length; GCL, genital cone length; GCW, genital cone width; EL, egg length; EW, egg width; OPL/PSL, ratio of opisthosoma to prosoma length; VSL/OSL, sucker length ratio; VSW/OSW, sucker width ratio; OSL/PHL, oral sucker to pharynx length ratio; diam., diameter. * dimensions were calculated from original descriptions

Table S2 Estimates of Average Evolutionary Divergence (d) and Standard error (s/e) over Sequence Pairs within Groups for the D2 domain of the 28S rDNA. Analyses were conducted using the Maximum Composite Likelihood model. This analysis involved 54 nucleotide sequences. Codon positions included were 1st+2nd+3rd+Noncoding. All positions with less than 95% site coverage were eliminated, i.e., fewer than 5% alignment gaps, missing data, and ambiguous bases were allowed at any position (partial deletion option). There were a total of 556 positions in the final dataset. Evolutionary analyses were conducted in MEGA11 (Tamura et al. 2021). The presence of n/c in the results denotes cases in which it was not possible to estimate evolutionary distances

	d	s/e
Strigea spp.	0,0152	0,0034
Parastrigea spp.	0,0085	0,0032
Nematostrigea spp.	n/c	n/c
Ichthyocotylurus spp.	n/c	n/c
Cotylurus spp.	0,0213	0,0043
Cardiocephaloides spp.	0,0152	0,0039
Australapatemon spp.	0,0127	0,0048
Apharyngostrigea spp.	0,0048	0,0024
Apatemon spp.	0,0327	0,0065

Table S3 Estimates of Evolutionary Divergence over Sequence Pairs between Groups for the D2 domain of the 28S rDNA. Standard error estimate(s) are shown above the diagonal (blue). Analyses were conducted using the Maximum Composite Likelihood model. This analysis involved 54 nucleotide sequences. Codon positions included were 1st+2nd+3rd+Noncoding. All positions with less than 95% site coverage were eliminated, i.e., fewer than 5% alignment gaps, missing data, and ambiguous bases were allowed at any position (partial deletion option). There were a total of 556 positions in the final dataset. Evolutionary analyses were conducted in MEGA11 (Tamura et al. 2021)

	**[1]**	**[2]**	**[3]**	**[4]**	**[5]**	**[6]**	**[7]**	**[8]**	**[9]**
**[1]** *Strigea*_spp.		0,0062	0,0123	0,0164	0,0162	0,0151	0,0109	0,0053	0,0085
**[2]** *Parastrigea*_spp.	0,0274		0,0115	0,0158	0,0156	0,0148	0,0114	0,0046	0,0087
**[3]** *Nematostrigea*_spp.	0,0675	0,0584		0,0130	0,0121	0,0120	0,0144	0,0122	0,0120
**[4]** *Ichthyocotylurus*_spp.	0,1001	0,0948	0,0688		0,0087	0,0125	0,0167	0,0159	0,0151
**[5]** *Cotylurus*_spp.	0,1008	0,0947	0,0653	0,0427		0,0107	0,0172	0,0157	0,0148
**[6]** *Cardiocephaloides*_spp.	0,0940	0,0904	0,0665	0,0646	0,0599		0,0169	0,0145	0,0155
**[7]** *Australapatemon*_spp.	0,0582	0,0619	0,0854	0,1054	0,1096	0,1098		0,0108	0,0089
**[8]** *Apharyngostrigea*_spp.	0,0223	0,0152	0,0645	0,0943	0,0938	0,0867	0,0555		0,0076
**[9]** *Apatemon*_spp.	0,0466	0,0469	0,0711	0,0963	0,0956	0,1037	0,0459	0,0386	

Table S4 Estimates of Average Evolutionary Divergence (d) and Standard error (s/e) over Sequence Pairs within species for the *cox*1 domain of the mDNA. Analyses were conducted using the Maximum Composite Likelihood model. This analysis involved 54 nucleotide sequences. Codon positions included were 1st+2nd+3rd+Noncoding. All positions with less than 95% site coverage were eliminated, i.e., fewer than 5% alignment gaps, missing data, and ambiguous bases were allowed at any position (partial deletion option). There were a total of 236 positions in the final dataset. Evolutionary analyses were conducted in MEGA11 (Tamura et al. 2021). The presence of n/c in the results denotes cases in which it was not possible to estimate evolutionary distances

	d	s.e
Strigea falconis	0,01	0,0045
Cotylurus syrius	0,0762	0,0158
Strigea strigis	0	0
Strigea vandenbrokae	0	0
Apatemon fuhrmanni	0,0064	0,0038
Strigea magnirostris	0	0
Strigea robusta (syn. *Parastrigea robusta*)	0,0225	0,0068
Strigea macrobursa (syn. *Parastrigea macrobursa*)	0,0073	0,0042

Table S5 Estimates of Evolutionary Divergence over Sequence Pairs between species for the *cox*1 domain of the mDNA. Standard error estimate(s) are shown above the diagonal (blue). Analyses were conducted using the Maximum Composite Likelihood model. This analysis involved 54 nucleotide sequences. Codon positions included were 1st+2nd+3rd+Noncoding. All positions with less than 95% site coverage were eliminated, i.e., fewer than 5% alignment gaps, missing data, and ambiguous bases were allowed at any position (partial deletion option). There were a total of 236 positions in the final dataset. Evolutionary analyses were conducted in MEGA11 (Tamura et al. 2021)

	**[1]**	**[2]**	**[3]**	**[4]**	**[5]**	**[6]**	**[7]**	**[8]**
**[1]** *Strigea_falconis*		0,045	0,044	0,049	0,037	0,031	0,055	0,059
**[2]** *Cotylurus_syrius*	0,209		0,033	0,038	0,037	0,046	0,035	0,036
**[3]** *Strigea_strigis*	0,206	0,151		0,033	0,042	0,042	0,028	0,037
**[4]** *Strigea_vandenbrokae*	0,208	0,190	0,158		0,036	0,042	0,038	0,043
**[5]** *Apatemon_fuhrmanni*	0,181	0,162	0,172	0,163		0,037	0,030	0,041
**[6]** *Strigea_magnirostris*	0,129	0,218	0,204	0,198	0,184		0,043	0,040
**[7]** *Strigea_robusta (syn. Parastrigea robusta)*	0,248	0,179	0,110	0,198	0,153	0,219		0,033
**[8]** *Strigea_macrobursa* (syn. *Parastrigea macrobursa*)	0,233	0,161	0,154	0,190	0,167	0,178	0,155	

Table S6 List of bird species recorded as prey of gyrfalcons during spring and summer in north-east Iceland in 1981–2023, analysis by OKN; only adult birds are included. Bird species recorded as hosts of metacercariae of *Strigea falconis* in literature (Sudarikov 1959, 1984; Bykhovskaya-Pavlovskaya 1962; Dubois 1968) are marked with an asterisk

Order	Bird species	Migratory	N examined	% of prey
**Anseriformes**	**Anatidae**			
	*Anas acuta**	yes	12	0.02
	*Anas crecca**	partly	222	0.42
	*Anas platyrhynchos**	partly	537	1.03
	Anas platyrhynchos domesticus	no	1	0.00
	*Anser anser**	partly	14	0.03
	Anser brachyrhynchus	yes	37	0.07
	Aythya fuligula	yes	824	1.57
	Aythya marila	yes	125	0.24
	Branta leucopsis	yes	2	0.00
	Bucephala islandica	no	167	0.32
	Clangula hyemalis	no	142	0.27
	Histrionicus histrionicus	no	403	0.77
	Mareca penelope	partly	3741	7.15
	*Mareca strepera**	yes	111	0.21
	Melanitta nigra	yes	35	0.07
	Mergus merganser	no	3	0.01
	Mergus serrator	no	110	0.21
	Somateria mollissima	no	6	0.01
	*Spatula clypeata**	yes	1	0.00
**Charadriiformes**	**Alcidae**			
	Alca torda	partly	128	0.24
	Alle alle	yes	1	0.00
	Cepphus grylle	no	26	0.05
	Fratercula arctica	yes	3826	7.31
	Uria aalge	no	16	0.03
	**Charadriidae**			
	*Charadrius hiaticula**	yes	2	0.00
	Pluvialis apricaria	yes	202	0.39
	**Haematopodidae**			
	Haematopus ostralegus	partly	2	0.00
	**Laridae**			
	*Chroicocephalus ridibundus**	partly	73	0.14
	Larus canus	partly	3	0.01
	*Larus fuscus**	yes	4	0.01
	Rissa tridactyla	yes	105	0.20
	Sterna paradisaea	yes	433	0.83
	**Scolopacidae**			
	Arenaria interpres	yes	2	0.00
	Calidris alpina	yes	12	0.02
	Calidris canutus	yes	1	0.00
	Calidris maritima	partly	2	0.00

	*Gallinago gallinago**	yes	272	0.52
	*Limosa limosa**	yes	94	0.18
	Numenius phaeopus	yes	1594	3.05
	*Phalaropus lobatus**	yes	5	0.01
	Scolopax rusticola	yes	2	0.00
	*Tringa totanus**	yes	28	0.05
	**Stercorariidae**			
	Stercorarius parasiticus	yes	22	0.04
**Columbiformes**	**Columbidae**			
	Columba livia	no	20	0.04
	Columba palumbus	yes	1	0.00
**Falconiformes**	**Falconidae**			
	Falco columbarius	partly	2	0.00
**Galliformes**	**Phasianidae**			
	Lagopus muta	no	38841	74.22
**Gruiformes**	**Rallidae**			
	*Fulica atra**	yes	1	0.00
**Passeriformes**	**Calcariidae**			
	Plectrophenax nivalis	partly	4	0.01
	**Corvidae**			
	Corvus corax	no	2	0.00
	**Motacillidae**			
	Anthus pratensis	yes	8	0.02
	*Motacilla alba**	yes	1	0.00
	**Muscicapidae**			
	Oenanthe oenanthe	yes	1	0.00
	**Turdidae**			
	Turdus iliacus	yes	41	0.08
	Turdus pilaris	yes	1	0.00
**Podicipediformes**	**Podicipedidae**			
	Podiceps auritus	partly	17	0.03
**Procellariiformes**	**Procellariidae**			
	Fulmarus glacialis	yes	36	0.07
**Strigiformes**	**Strigidae**			
	*Asio flammeus**	partly	5	0.01
	**Total number**		**52329**	**100.00**

References to Table S1 and S6

Bykhovskaya-Pavlovskaya IE (1962) The trematode fauna of birds of the USSR. An ecological and geographical account. Izdatelstvo Akademii Nauk SSSR, Moscow, Leningrad. (in Russian)

Drago FB, Núñez V, Lunaschi LI (2018) Strigeid parasites of *Circus buffoni* from Argentina, with the description of a new species of *Parastrigea* Szidat, 1928. Par Res 117:751–758. https://doi.org/10.1007/s00436-017-5746-x

Dubois G (1938) Monographie des Strigeida (Trematoda). Mém Soc Neuchateloise Sci Nat 6:1–503.

Dubois G (1968) Synopsis des Strigeidae et des Diplostomatidae (Trematoda). Mém Soc Neuchateloise Sci Nat 10:1–259.

Dubois G, Rausch RL (1950) A contribution to the study of North American strigeids (Trematoda). Am Midl Nat 43:1–31.

Dubois G, Rausch RL (1965) Studies on the helminth fauna of Alaska. XLIII. *Strigea macropharynx* sp. n., a trematode parasite of *Falco rusticolus* L. J Parasitol 51:770–772. https://doi.org/10.2307/3276154

Heneberg P, Sitko J, Těšínský M, Rząd I, Bizos J (2018) Central European Strigeidae Railliet, 1919 (Trematoda: Strigeidida): Molecular and comparative morphological analysis suggests the reclassification of *Parastrigea robusta* Szidat, 1928 into *Strigea* Abildgaard, 1790. Parasitol Int 67:688–701. https://doi.org/10.1016/j.parint.2018.07.003. Epub 2018 Jul 9. PMID: 30003965

López-Jiménez A, González-García M, Andrade-Gómez L, García-Varela M (2023) Phylogenetic analyses based on molecular and morphological data reveal a new species of *Strigea* Abildgaard, 1790 (Digenea: Strigeidae) and taxonomic changes in strigeids infecting Neotropical birds of prey. J Helminthol 97:E35. https://doi.org/10.1017/S0022149X23000196

Lunaschi LI, Drago FB (2006) Strigeid parasites of the roadside hawk, *Buteo magnirostris* (Aves: Falconiformes), from Argentina. Zootaxa 1106:25–33.

Sudarikov V E (1959) Order Strigeidida (La Rue, 1926) Sudarikov, 1959. In: Skrjabin KI (ed) Trematodes of Animals and Man. Osnovy trematodologii, Izdatelstvo AN SSSR, Moscow, 16: 217–631. (in Russian)

Sudarikov VE (1984) Trematodes of the fauna of the USSR. Izdatelstvo Nauka, Moscow. (in Russian)
